# The Relationships between HIV-1 Infection, History of Methamphetamine Use Disorder, and Soluble Biomarkers in Blood and Cerebrospinal Fluid

**DOI:** 10.3390/v13071287

**Published:** 2021-07-01

**Authors:** T. Jordan Walter, Jennifer Iudicello, Debra Rosario Cookson, Donald Franklin, Bin Tang, Jared W. Young, William Perry, Ronald Ellis, Robert K. Heaton, Igor Grant, Arpi Minassian, Scott Letendre, on behalf of the Translational Methamphetamine AIDS Research Center (TMARC)

**Affiliations:** 1 Department of Psychiatry, University of California San Diego, 9500 Gilman Drive, La Jolla, CA 92093, USA; jiudicello@health.ucsd.edu (J.I.); drosario@health.ucsd.edu (D.R.C.); dofranklin@health.ucsd.edu (D.F.); bit001@health.ucsd.edu (B.T.); jaredyoung@ucsd.edu (J.W.Y.); wperry@health.ucsd.edu (W.P.); rheaton@health.ucsd.edu (R.K.H.); igrant@health.ucsd.edu (I.G.); aminassian@health.ucsd.edu (A.M.); 2 Department of Neurosciences, University of California San Diego, 9500 Gilman Drive, La Jolla, CA 92093, USA; roellis@health.ucsd.edu; 3 VA Center of Excellence for Stress and Mental Health, Veterans Administration San Diego Healthcare System, 3350 La Jolla Village Drive, San Diego, CA 92161, USA; 4 Division of Infectious Disease and Global Public Health, Department of Medicine, University of California San Diego, 9500 Gilman Drive, La Jolla, CA 92093, USA

**Keywords:** HIV, methamphetamine, substance use disorder, immune, inflammation, biomarker, cognition

## Abstract

Methamphetamine (METH) use disorder is highly prevalent among people with HIV (PWH) and is a significant public health problem. HIV and METH use are each associated with immune system dysfunction; however, the combined effects on the immune system are poorly understood. This cross-sectional project measured soluble immune biomarkers in plasma and cerebrospinal fluid (CSF) collected from a control group, people with a history of a METH use disorder (METH+), PWH with no history of METH use disorder (HIV+), and PWH with a history of METH use disorder (HIV+/METH+). HIV, METH, and immune dysfunction can also be associated with affective and cognitive deficits, so we characterized mood and cognition in our participants. Two factor analyses were performed for the plasma and CSF biomarkers. Plasma IL-8, Ccl2, VEGF, and 8-isoprostane loaded onto one factor that was highest in the HIV+/METH+ group (*p* < 0.047) reflecting worse inflammation, vascular injury, and oxidative stress. This plasma factor was also negatively correlated with delayed recall (R = −0.49, *p* = 0.010), which was worst in the HIV+/METH+ group (*p* = 0.030 compared to the control group). Overall, these data implicate that combined HIV-1 infection and METH use may exacerbate inflammation, leading to worse cognition.

## 1. Introduction

Methamphetamine (METH) use disorder is highly prevalent among people with HIV (PWH) and is a significant public health concern. One study found that 13% of PWH have a current METH use disorder [1]. Another found that 31% of outpatient PWH used METH in the past year [2]. Both HIV and METH alter immune system function. For example, even in PWH on anti-retroviral therapy (ART) for one year, the pro-inflammatory cytokine TNFα remains elevated compared to people without HIV [3]. PWH on ART also have evidence of widespread neuroinflammation compared to people without HIV [4]. METH also influences immune biomarkers in the circulatory and central nervous systems (CNS) [5] and contributes to oxidative stress [6]. For example, IL-6 is elevated in the plasma of people with METH dependence compared to healthy controls [7]. Animal studies found that METH administration increases TNFα [8] and IL-1β [9] in the brain. Neuroinflammation can persist in METH users even in sustained abstinence [10]. These changes in the immune system are important as they are associated with—and may be causally related to—negative changes in affect and cognition [11].

Little is known about the combined effects of HIV and METH on the immune system and how immune changes may be related to affect or cognitive function in HIV+/METH+ people. One study found the combination of the HIV protein, Tat, and METH treatment synergistically increased TNFα and IL-1β in the brains of animals [12]. In contrast, no interactive effects of HIV and METH on plasma cytokines were observed in humans, although the sample size in this study was small and the number of cytokines examined was limited [13]. Previous studies also show that HIV and METH impair cognitive function more than either alone [14,15]. To our knowledge, no previous studies have examined the combined effects of HIV and METH on central immune function. Overall, the relationships between HIV, METH, the immune system, and potential associations with mood and cognition remain poorly understood.

The objective of this project was to determine the relationship between HIV-1 infection (hereafter referred to simply as “HIV”) and a history of METH use disorder on soluble biomarkers in the plasma and cerebrospinal fluid (CSF) and their relationship to affect and cognition. Biomarkers were selected based on our previous findings, as well as literature that linked these biomarkers to HIV, METH, or brain injury [16,17]. This included immune, oxidative stress, vascular, and neuronal biomarkers. We also examined affect (e.g., depression, anxiety) and cognitive performance across seven domains (e.g., attention, memory), hypothesizing that combined HIV and METH would be associated with worse inflammation, worse affect, and worse cognition. Such findings would provide insights into mechanisms of pathology in combined HIV and METH use and potentially inform preventative or therapeutic strategies.

## 2. Materials and Methods

The University of California San Diego’s (UCSD) Translational Methamphetamine AIDS Research Center (TMARC; P50DA026306) is a multidisciplinary research program funded by the National Institute of Drug Abuse that focuses on understanding the combined effects of HIV and METH use disorders on brain structure and function. The UCSD Human Research Protections Program approved the project and all participants provided written informed consent for the procedures.

### 2.1. Human Participants

Four groups of participants were assessed: HIV-/METH-, HIV-/METH+, HIV+/METH-, and HIV+/METH+. HIV-1 status was determined by enzyme-linked immunosorbent assay (ELISA) and a confirmatory Western blot. Participants in the METH+ groups met Diagnostic and Statistical Manual-Fourth Edition (DSM-IV) criteria (as assessed by the Composite International Diagnostic Interview Version 2.1) for both lifetime METH dependence and METH abuse or dependence within the past 18 months.

Potential participants were excluded if they met DSM-IV diagnostic criteria for current abuse or dependence on alcohol or drugs other than METH. Potential participants were also excluded if they reported neurological (e.g., seizure disorder, stroke with residual sequelae) or psychiatric (e.g., psychosis) conditions that were sufficiently severe to confound attribution of the neuropsychological testing results to HIV or METH. PWH were excluded if the HIV RNA in plasma exceeded 1000 copies/mL. People with untreated infections other than HIV that can affect the central nervous system (e.g., syphilis, hepatitis C) were also excluded. A urine drug screen was performed at the time of testing (Biotechnostix; Markham, ON, Canada) and those who tested positive for METH or any other drugs were excluded from our analyses because of the potential influence of intoxication on the examined biomarkers.

### 2.2. Plasma and Cerebrospinal Fluid Biomarker Sampling and Measurement

Blood plasma for biomarker assays was collected by phlebotomy using EDTA vacuum tubes. Whole blood was centrifuged at room temperature and plasma was aliquoted for storage at −80 °C. CSF was collected by lumbar puncture using a non-traumatic spinal needle and aseptic technique. CSF was centrifuged at low speed to separate cells. Supernatant was aliquoted and stored at −80 °C until the time of the assays. Protein biomarkers for this analysis were measured using commercially available immunoassays according to the instructions of the manufacturer. C-reactive protein (CRP), soluble ICAM-1, IL-6, IL-8, CXCL10, Ccl2, soluble TNFR-II, and VEGF were measured using electrochemical luminescence immunoassays (MesoScale Discovery, Rockville, MD, USA). The remainder of the biomarkers were measured using a quantitative sandwich enzyme immunoassay (8-isoprostane: Cayman, Ann Arbor, MI, USA; 8-oxo-2′-deoxyguanosine (8-oxo-dG): Trevigen, Gaithersburg, MD, USA; D-dimer: Biomedica, Windsor, ON, Canada; malondialdehyde (MDA): Cell Biolabs, San Diego, CA, USA; neurofilament-light (NFL): Tecan, Männedorf, Switzerland; soluble CD14 and uPAR: R&D Systems, Minneapolis, MN, USA). All assays were performed in duplicate. Assays were repeated when the coefficient of variation was greater than 20%. In addition, 10% of all assays were repeated to assess operator and batch consistency. To further limit the influence of assay variation, raw biomarker values were transformed to plate-normalized Z-scores (i.e., Z-scores were calculated for each biomarker sample within a plate).

### 2.3. Affect Assessment and Neuropsychological Testing

Depressive symptoms were assessed using Beck’s Depression Inventory (BDI)—2nd Edition. Anxiety was assessed using the Profile of Mood States (POMS). The neurocognitive assessment included an estimate of premorbid cognitive ability (i.e.,—the reading subtest of the Wide Range Achievement Test-4 (WRAT-4)) [18] and neurocognitive tests assessing the following seven domains commonly affected by HIV and/or METH: verbal fluency (letter fluency, animal fluency, and verb fluency), executive functioning (Trail Making Test Part B, the Wisconsin Card Sorting Test-64, and the Stroop Color-Word Trial), speed of information processing (Wechsler Adult Intelligence Scale-III Digit Symbol and Symbol Search subtests, Stroop Color Naming Trial, and Trail Making Test Part A), learning (Hopkins Verbal Learning Test-Revised (HVLT-R) and Brief Visual Memory Test-Revised (BVMT-R)), memory (delayed recall of HVLT-R and BVMT-R), working memory (Paced Auditory Serial Addition Test-50 and Wechsler Memory Scale III Spatial Span), and complex motor skills (grooved pegboard). Raw scores were converted to T-scores using published, demographically adjusted normative standards for age, education, sex, and race/ethnicity [19,20].

### 2.4. Data Processing and Statistical Analyses

Statistical analyses were conducted using the Statistical Package for the Social Sciences (SPSS) version 26. When a biomarker distribution remained skewed after Z-score normalization, values were log-transformed to improve distribution symmetry.

Two types of analyses were performed on the log-transformed Z-scores. The first analysis consisted of 2 × 2 analyses of covariance (ANCOVAs) that included age and sex as covariates (both of which were significantly different between the four groups) to determine the main effects of HIV and METH, as well as interactions. Significant interactions were followed-up with Sidak’s post-hoc test to correct for multiple comparisons while examining differences between individual groups. The second analysis consisted of a factor analysis. Factor analysis was performed on the log-transformed Z-scores of the biomarkers to reduce the dimensionality of the data and identify patterns that would not be evident by analysis of individual biomarkers. Separate factor analyses were performed for the plasma and CSF biomarkers. Varimax rotations were applied to the data and a lower limit to absolute loading values was applied such that each biomarker was loaded onto only one factor. Factors with eigenvalues greater than one were saved for subsequent analyses. Two-by-two ANCOVAs were performed on each of the resulting factors with age and sex included as covariates. The main effects of HIV, METH, and interactions were assessed as described above.

For analysis of the cognitive and affective measures, 2 × 2 ANOVAs were performed as described above. For the cognitive measures, we used the mean T-scores on the tests described above. For the correlation analyses, we were only interested in changes that were unique to the combination of HIV and METH; therefore, we only focused on endpoints for which there was an additive or synergistic change with both HIV and METH. For the correlation analysis, Spearman rank correlations were performed because not all measures were normally distributed. Correlational analyses were performed within groups but not across all groups. Statistical significance for all analyses was defined as *p* < 0.05.

## 3. Results

### 3.1. Demographics

Data were collected from a total of 146 participants, 46 of whom had biomarkers measured in paired plasma and CSF samples collected within an hour, 21 of whom had biomarkers measured only in CSF, and 79 of whom had biomarkers measured only in plasma. Since some participants did not have paired CSF and plasma samples, two sets of demographic information were calculated: one for the participants with plasma samples (*n* = 125, Table 1) and one for participants with CSF samples (*n* = 67, Table S1). For participants with plasma samples, groups differed in age, with the METH+ groups being younger than METH- groups, and in sex, with the HIV+ groups having more men (Table 1). These groups also differed in education. Finally, the two HIV+ groups differed in estimated duration of infection. The groups were, however, well-matched for all other HIV illness and METH use characteristics (Table 1). See the Supplementary Materials for the demographic information on participants with CSF samples.

### 3.2. Concentrations of Individual Plasma and CSF Biomarkers

In plasma, IL-8, CXCL10, Ccl2, and sCD14 were higher in the HIV+ groups compared to the HIV- groups (*p* = 0.008, *p* < 0.0005, *p* = 0.014, and *p* = 0.008, respectively) (Table S2). Plasma IL-6 was lower in the METH+ groups compared to the METH- groups (*p* = 0.029) (Table S2). In CSF, 8-isoprostane and IL-8 levels were higher in the HIV+/METH- group compared to the HIV-/METH- group (*p* = 0.017 and *p* = 0.042, respectively) (Table S3). Additionally, CXCL10 was higher in the HIV+ groups compared to the HIV- groups (*p* = 0.008) (Table S3).

### 3.3. Factor Analysis of Plasma and CSF Biomarkers

**Figure 1:**
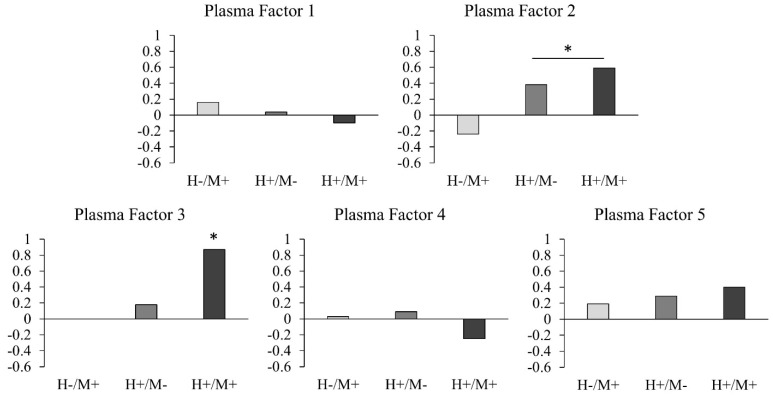
Effect sizes of each group compared to the HIV-/METH- group for the plasma factors. Effect sizes (using Cohen’s d) were calculated for the HIV-/METH+, HIV+/METH-, and HIV+/METH+ group compared to the HIV-/METH- group for each factor. * Significant main effect of HIV for Plasma Factor 2 (i.e., HIV+ groups > HIV- groups) and significant group differences for HIV+/METH+ group for Plasma Factor 3 (i.e., HIV+/METH+ group > all other groups).

Two separate factor analyses were performed for the plasma and CSF biomarkers. The plasma factor analysis identified five factors (Table 2). Plasma Factors 1, 2, 3, 4, and 5 had eigenvalues of 3.72, 1.64, 1.19, 1.05, and 1.03 and accounted for 26.6%, 11.8%, 8.5%, 7.5%, and 7.3% of the variance, respectively. CXCL10 and sTNFR2 (both associated with inflammation), and ICAM1 and uPAR (both associated with vascular injury), loaded onto Plasma Factor 2, which was higher in the HIV+ groups compared to the HIV- groups (*p* = 0.005, Cohen’s d = 0.52) (Figure 1). IL-8 and Ccl2 (both associated with inflammation), VEGF (associated with angiogenesis), and 8-isoprostane (associated with oxidative stress) loaded onto Plasma Factor 3, which was also higher in the HIV+ groups compared to the HIV- groups (main effect of HIV: *p* = 0.011, Cohen’s d = 0.48). Plasma Factor 3 was also higher in the HIV+/METH+ compared to all other groups (*p* ≤ 0.047, Cohen’s d = 0.87 compared to the HIV-/METH- group) (Figure 1). There were no group differences or interactions for Plasma Factors 1, 4, or 5.

The CSF factor analysis resulted in four factors (Table S4). CSF Factors 1, 2, 3, and 4 had eigenvalues of 5.32, 1.54, 1.27, and 1.16 and accounted for 35.5%, 10.3%, 8.5%, and 7.8% of the variance, respectively. There were no effects of HIV or METH, or HIV-by-METH interactions, for any of the four CSF factors (see the Supplementary Materials).

### 3.4. Correlations with Affective and Cognitive Measures

**Figure 2:**
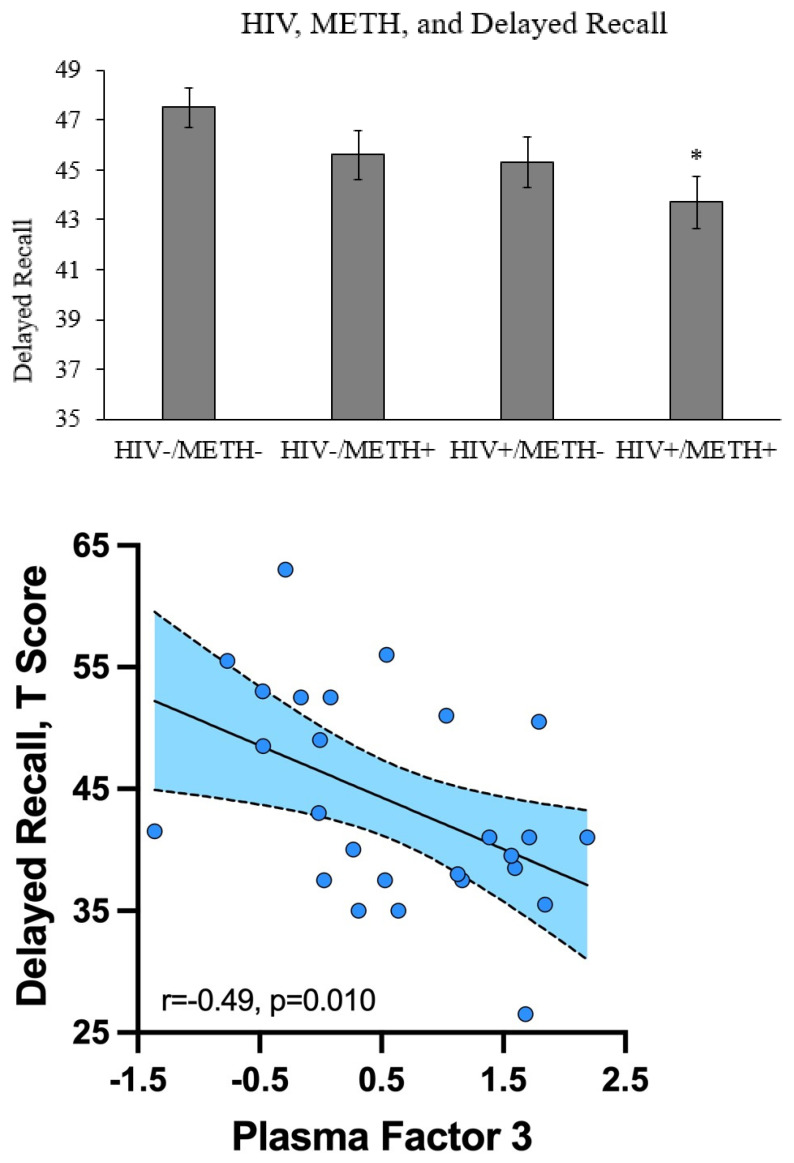
The relationship between HIV, METH, Plasma Factor 3, and delayed recall. Delayed recall was measured in each of the four groups. * = *p* < 0.05 compared to the HIV-/METH- group. Delayed recall negatively correlated with Plasma Factor 3 in the HIV+/METH+ group, but not any of the other three groups.

We examined how HIV and METH related to depressive symptoms (as measured by the BDI-II), anxiety symptoms (as measured by the tension/anxiety subscale of the POMS), and seven cognitive domains (verbal fluency, executive functioning, speed of information processing, learning, delayed recall, working memory, and complex motor skills). Combined HIV and METH was associated with significantly lower delayed recall compared to the HIV-/METH- group (*p* = 0.030, Cohen’s d = −0.42) (Figure 2). There were no additive or synergistic effects of combined HIV and METH for any other measure.

Because we were particularly interested in the combined effects of HIV and METH, we focused on measures that had evidence of additive or synergistic effects in our correlation analyses. Only two measures met this criterion: Plasma Factor 3 and delayed recall. A correlation analysis showed that Plasma Factor 3 was significantly negatively correlated with delayed recall in the HIV+/METH+ group (Spearman’s rho: −0.49, *p* = 0.010) (Figure 2). Plasma Factor 3 and delayed recall were not significantly correlated in any of the other three groups, suggesting the relationship may be unique to people with both HIV and METH use disorder.

## 4. Discussion

In this cross-sectional study of the relationship between HIV, lifetime METH use disorder, and soluble plasma and CSF biomarkers, we found that HIV and METH together were associated with a combination of soluble immune (IL-8, Ccl2), vascular (VEGF), and oxidative stress (8-isoprostane) plasma biomarkers. This combination of plasma biomarkers negatively correlated with delayed recall, which was lowest in the HIV+/METH+ group. We also identified several biomarkers that were higher in the plasma and CSF of PWH. A history of METH use disorder was only clearly associated with lower plasma IL-6.

### 4.1. Significance of Findings

This study was, to our knowledge, the first to examine the combined effects of HIV and METH on both plasma and CSF immune biomarkers. The primary novel finding was that the combination of HIV and METH use disorder was associated with higher values of a plasma factor that included IL-8, VEGF, Ccl2, and 8-isoprostane. This result is consistent with the hypothesis that HIV and METH together may have greater effects on the immune system than either condition alone. IL-8 is a chemokine that promotes acute inflammation by recruiting and activating neutrophils [21]. Like IL-8, Ccl2 also promotes inflammation by recruiting leukocytes to sites of injury [22]. VEGF is involved in angiogenesis [23] and, in the context of inflammation, increases vascularity at the site of inflammation, causing the reaction to be more severe [24]. Lastly, 8-isoprostane is a biomarker of oxidative stress [25]. It is worth noting the increase in inflammatory biomarkers and the associated decrease in memory occurs in an HIV+ population mostly treated with ART.

Although interpretation of the factors resulting from factor analysis is not always straightforward, the fact that Plasma Factor 3 was highest in the HIV+/METH+ group may imply that an inflammatory process is exacerbated in people with both HIV and METH use disorder. For example, cytokines such as IL-17 [26] or signaling through Toll-like receptor 4 [27] promote the production of the biomarkers that loaded onto our Plasma Factor 3. Upregulated activity of an inflammatory transcription factor such as NF-κB or JAK/STAT may also underlie the production of these biomarkers. Other studies have reported that the biomarkers that load onto our Plasma Factor 3 are biologically interrelated. For example, 8-isoprostane increases the expression of IL-8 [28], supporting the biological plausibility of this factor. Interestingly, Plasma Factor 3 was negatively correlated with delayed recall. Many prior studies found that increased inflammation in PWH was associated with poorer cognitive performance [29,30]. Since our Plasma Factor 3 was associated with several immune biomarkers, we speculated that this factor represents inflammation-associated processes that contribute to poor delayed recall.

Many chronic inflammatory conditions ranging from hepatitis C [31,32] to rheumatoid arthritis [33] are associated with cognitive impairment. Inflammation-driven cognitive decline may include multiple, related biological mechanisms, including generation of reactive oxygen species (ROS). ROS damage the lipids, proteins, and carbohydrates that make up the cellular machinery, thereby weakening neuronal function. Moreover, ROS promote apoptosis, resulting in neuronal death [34]. Inflammation also promotes vascular dysfunction, including vascular leakage and arterial narrowing and stiffening, all of which impair cerebral blood flow and impair neuronal function [35]. Finally, neuroinflammation and activation of microglia can promote the release of inflammatory cytokines that can trigger apoptosis in neurons [36]. These may all be mechanisms by which inflammation in PWH who use METH contributes to memory decline.

Other patterns were present in our data even if they did not all reach statistical significance. Some biomarkers or factors tended to be higher in PWH but lower in people with both HIV and METH use disorder, for example, CSF Factor 3 or CSF CRP. This pattern may be due to the fact that HIV activates some immune responses, but METH use alters the process in an unexpected manner. Such a pattern has been noted in some in vitro studies examining hepatitis C in which METH had an immunosuppressant effect in HCV-infected hepatocytes [37]. METH may enhance some immune processes but inhibit others [38]. Other patterns observed include an additive effect of combined HIV and METH, such as with CSF Factor 4. Overall, the diverse patterns of biomarker and factor responses suggest the combination of HIV and METH has complex effects on the immune system that require further investigation using larger sample sizes in longitudinal and interventional designs.

### 4.2. Strengths and Limitations of This Study

Strengths of this study include the large number of biomarkers assessed and the fact they were assessed in plasma and CSF. To our knowledge, this study is the first to examine the combined effects of HIV and METH on soluble immune biomarkers in the CSF. This study also involved phenotyping of human participants that allowed for correlations of immune dysfunction with affect and cognition. Limitations of the study include the relatively small number of CSF samples and the relatively small number of female participants, the latter of which limits the generalizability of our findings. Furthermore, there was a lack of matching on demographic characteristics such as age in this study. Although not ideal from a statistical standpoint, age differences reflect the demographics of the underlying populations, with METH users typically being younger than METH non-users. There was also substantial heterogeneity in the METH use patterns of subjects with a history of METH use disorder. Moreover, the plasma factors that had a significant relationship with HIV or METH (Plasma Factor 2 and Plasma Factor 3) had relatively low eigenvalues (1.64 and 1.19) and accounted for relatively low variance (11.8% and 8.5%). Finally, as a cross-sectional study, we cannot draw conclusions regarding causality from our data.

### 4.3. Practical Implications

Our results suggest that combined HIV and a history of METH use disorder have complex effects on soluble biomarkers. METH may exacerbate the immune processes that are affected by HIV and may contribute to memory decline. This observation underscores that METH use in PWH may be especially harmful and should concern patients and physicians alike. Anti-inflammatory therapies may be useful to help reverse or prevent cognitive impairment in PWH who use METH; however, more research is needed. Targeting IL-8, Ccl2, VEGF, and 8-isoprostane may be beneficial.

### 4.4. Directions for Future Study

Future studies might investigate whether there is a causal link between inflammation and cognition in PWH and METH use disorder by examining whether interventions like anti-inflammatory therapies alter soluble biomarkers and cognition in this population. Such research would help determine if the relationships we observed are causal and reversible. Future studies could also identify whether other elements of biological pathways, such as transcription factors or immune receptors, are involved and could be targeted.

## 5. Conclusions

This study examined the relationship between HIV, METH use disorder, soluble biomarkers in the plasma and CSF, mood, and cognition. We found that the combination of HIV and METH was associated with higher levels of immune, vascular, and oxidative stress biomarkers in the blood, as well as with worse memory. The findings support strengthening ongoing efforts to reduce or eliminate METH use among PWH and to identify new therapies that will prevent or treat these complications.

## Figures and Tables

**Table 1:** Demographic information for participants with plasma samples.

	HIV-/METH- (*n* = 52)	HIV-/METH+ (*n* = 16)	HIV+ /METH- (*n* = 31)	HIV+ /METH+ (*n* = 26)	*p* Value
Age (years)	46.0 ± 17.0	39.1 ± 14.1	47.0 ± 14.8	40.0 ± 7.0	0.013
Sex (%)	M: 48.1; F: 51.9	M: 62.5; F: 37.5	M: 87.1; F: 12.9	M: 96.1; F: 3.8	<0.0005
Ethnicity (%)	Asian: 5.8; Afr Am: 15.4; Hisp: 23; Other: 0; Cauc: 55.8	Asian: 6.2; Afr Am: 25.0; Hisp: 18.7; Other: 0; Cauc: 50.0	Asian: 0; Afr Am: 6.5; Hisp: 29.0; Other: 3.2; Cauc: 61.3	Asian: 0; Afr Am: 3.8; Hisp: 38.4; Other: 3.8% Cauc: 53.8	NS
Education (years)	15.0 ± 2.0	12.2 ± 2.8	14.4 ± 2.4	14.2 ± 2.1	0.024
Age at first METH use (years)	-	24.5 ± 12.8	-	25.7 ± 8.0	NS
Days since last METH use (days)	-	260 ± 524	-	168 ± 169	NS
Total quantity METH use (grams)	-	6346 ± 11,530	-	2382 ± 2691	NS
Current CD4+ cell count (cells/uL)	-	-	750 ± 309	665 ± 257	NS
Estimated duration of HIV infection (years)	-	-	13.1 ± 10.1	8.0 ± 5.5	0.025
Plasma HIV RNA < 50 copies/mL (%)	-	-	83.9	84.6	NS
Current ART use (%)	-	-	90.3	100	NS

Data are presented as mean ± standard deviation unless otherwise noted. NS = not significantly different; ART: Anti-retroviral therapy.

**Table 2:** Factor loadings of plasma biomarkers.

	Factor 1	Factor 2	Factor 3	Factor 4	Factor 5
CRP	0.843				
IL-6	0.730				
D-dimer	0.581				
CXCL10		0.753			
sTNFR2		0.694			
ICAM1		0.635			
uPAR		0.566			
IL-8			0.737		
VEGF			0.713		
Ccl2			0.671		
8-isoprostane			0.532		
8-oxo-dG				0.781	
MDA				0.621	
sCD14					0.834

## Supplementary Materials

The following are available online at https://www.mdpi.com/article/10.3390/v13071287/s1, Figure S1. Effect Sizes of Each Group Compared to the HIV-/METH- Group for Each Plasma Factor, Table S1. Demographic Information for Participants with CSF Samples, Table S2. Summary of Plasma Biomarker Concentrations, Table S3. Summary of CSF Biomarker Concentrations, Table S4. Factor Loadings of CSF Biomarkers.
