# Supplementary material for: The Relationships between HIV-1 Infection, History of Methamphetamine Use Disorder, and Soluble Biomarkers in Blood and Cerebrospinal Fluid
Source: Viruses. 2021 Jul 1;13(7):1287. doi: 10.3390/v13071287 (PMC8310127; doi:10.3390/v13071287)
Supplement: Supplementary file 1 [file viruses-13-01287-s001.zip › viruses-1273377-SI.pdf]

viruses

MDPI

Supplementary

Table S1. Demographic Information for Participants with CSF Samples.

	HIV-/METH- (*n*=18)	HIV-/METH+ (*n*=14)	HIV+ /METH- (*n*=22)	HIV+ /METH+ (*n*=13)	*P* value
Age (years)	44.9 ± 16.2	38.4 ± 12.1	47.7 ± 16.4	38.3 ± 8.3	**0.029**
Sex (%)	M: 61.1; F: 38.9	M: 64.3; F: 35.7	M: 95.4; F: 4.5	M: 92.3; F: 7.8	**0.016**
Ethnicity (%)	Asian: 5.6 Afr Am: 11.1; Hisp: 33.3; Other: 0 Cauc: 50.0	Asian: 0 Afr Am: 21.4; Hisp: 7.1; Other: 14.3; Cauc: 57.1	Asian: 0 Afr Am: 18.2; Hisp: 18.2; Other: 9.1; Cauc: 54.5	Asian: 0; Afr Am: 7.7; Hisp: 69.2; Other: 0; Cauc: 23.1	NS
Education (years)	14.9 ± 1.6	12.9 ± 2.7	14.7 ± 2.4	13.9 ± 2.5	**0.018**
Age First METH use (years)	-	25.0 ± 11.4	-	24.6 ± 6.7	NS
Days since last METH use (days)	-	276 ± 563	-	93 ± 126	NS
Total quantity METH use (grams)	-	3654 ± 5507	-	2269 ± 2056	NS
Current CD4+ Cell Count (cells/uL)	-	-	801 ± 307	651 ± 250	NS
Estimated Duration HIV Infection (years)	-	-	15.1 ± 10.6	7.4 ± 5.2	**0.021**
Plasma HIV RNA <50 copies/mL (%)	-	-	95.5	84.6	NS
Current ART Use (%)	-	-	95.5	100	NS

Data are presented as mean ± standard deviation unless otherwise noted. NS = not significantly different; ART: Antiretroviral therapy.

Table S2. Summary of Plasma Biomarker Concentrations.

	HIV-/METH-	HIV-/METH+	HIV+ /METH-	HIV+ /METH+	*P* value	Risk (Cohen's d)
8-iso-prostane	0.53 ± 0.09	0.56 ± 0.10	0.58 ± 0.08	0.59 ± 0.09	NS	
8-oxodG	0.63 ± 0.10	0.62 ± 0.06	0.65 ± 0.11	0.61 ± 0.09	NS	
CRP	0.20 ± 0.10	0.23 ± 0.12	0.26 ± 0.15	0.18 ± 0.12	NS	
D-dimer	0.36 ± 0.13	0.34 ± 0.15	0.37 ± 0.12	0.37 ± 0.15	NS	
ICAM1	0.45 ± 0.13	0.49 ± 0.11	0.48 ± 0.12	0.49 ± 0.14	NS	
IL-6	0.35 ± 0.10	0.36 ± 0.11	0.41 ± 0.14	0.32 ± 0.11	**0.029**	**METH: d= -0.36**
IL-8	0.38 ± 0.16	0.36 ± 0.12	0.44 ± 0.13	0.47 ± 0.13	**0.008**	**HIV: d=0.47**
CXCL10	0.30 ± 0.12	0.28 ± 0.11	0.38 ± 0.15	0.40 ± 0.14	**<0.0005**	**HIV: d=0.68**
Ccl2	0.36 ± 0.15	0.36 ± 0.15	0.44 ± 0.17	0.48 ± 0.13	**0.014**	**HIV: d=0.40**
MDA	0.44 ± 0.12	0.42 ± 0.14	0.44 ± 0.10	0.41 ± 0.08	NS	
sCD14	0.48 ± 0.15	0.46 ± 0.11	0.54 ± 0.12	0.52 ± 0.13	**0.006**	**HIV: d=0.45**
sTNFR2	0.41 ± 0.15	0.42 ± 0.10	0.44 ± 0.15	0.46 ± 0.12	NS	
uPAR	0.43 ± 013	0.44 ± 0.15	0.46 ± 0.16	0.40 ± 0.11	NS	
VEGF	0.37 ± 0.14	0.36 ± 0.15	0.39 ± 0.14	0.43 ± 0.15	NS	

Values represent the mean ± S.E.M. of the log transformed Z-scores of the raw biomarker data.

Table S3. Summary of CSF Biomarker Concentrations.

	HIV-/METH-	HIV-/METH+	HIV+ /METH-	HIV+ /METH+	*P* value	Risk (Cohen's d)
8-iso-prostane	0.31 ± 0.17	0.40 ± 0.16	0.45 ± 0.15	0.38 ± 0.11	**0.017**	**H+/M- > H-/M-: d=0.80**
8-oxodG	0.37 ± 0.17	0.33 ± 0.12	0.37 ± 0.16	0.33 ± 0.16	NS	
CRP	0.29 ± 0.10	0.29 ± 0.21	0.35 ± 0.17	0.27 ± 0.09	NS	
D-dimer	0.43 ± 0.13	0.45 ± 0.13	0.43 ± 0.13	0.41 ± 0.10	NS	
ICAM1	0.28 ± 0.15	0.31 ± 0.16	0.38 ± 0.18	0.31 ± 0.14	NS	

IL6	0.43 ± 0.11	0.47 ± 0.14	0.43 ± 0.14	0.39 ± 0.09	NS	
IL8	0.40 ± 0.14	0.44 ± 0.14	0.52 ± 0.12	0.44 ± 0.10	**0.042**	**H+/M- > H-/M-: d=0.40**
CXCL10	0.27 ± 0.09	0.28 ± 0.14	0.38 ± 0.15	0.34 ± 0.17	**0.008**	**HIV: d=0.60**
Ccl2	0.42 ± 0.17	0.42 ± 0.14	0.44 ± 0.15	0.41 ± 0.13	NS	
MDA	0.28 ± 0.15	0.32 ± 0.11	0.33 ± 0.14	0.32 ± 0.11	NS	
NFL	0.38 ± 0.13	0.35 ± 0.15	0.44 ± 0.18	0.34 ± 0.08	NS	
sCD14	0.32 ± 0.12	0.34 ± 0.18	0.41 ± 0.19	0.32 ± 0.11	NS	
sTNFR2	0.38 ± 0.14	0.39 ± 0.08	0.45 ± 0.16	0.39 ± 0.12	NS	
uPAR	0.33 ± 0.16	0.32 ± 0.13	0.41 ± 0.19	0.37 ± 0.08	NS	
VEGF	0.40 ± 0.14	0.52 ± 0.13	0.48 ± 0.15	0.44 ± 0.14	NS	

Values represent the mean ± S.E.M. of the log transformed Z-scores of the raw biomarker data.

Table S4. Factor Loadings of CSF Biomarkers.

	**Factor 1**	**Factor 2**	**Factor 3**	**Factor 4**
ICAM1	0.831			
uPAR	0.828			
sTNFR2	0.796			
sCD14	0.739			
NFL	0.671			
IL8	0.663			
CXCL10	0.536			
CRP	0.491			
IL6		0.825		
Ccl2		0.681		
D-dimer			0.804	
8-oxodG			0.696	
MDA				0.815
8iso				0.617

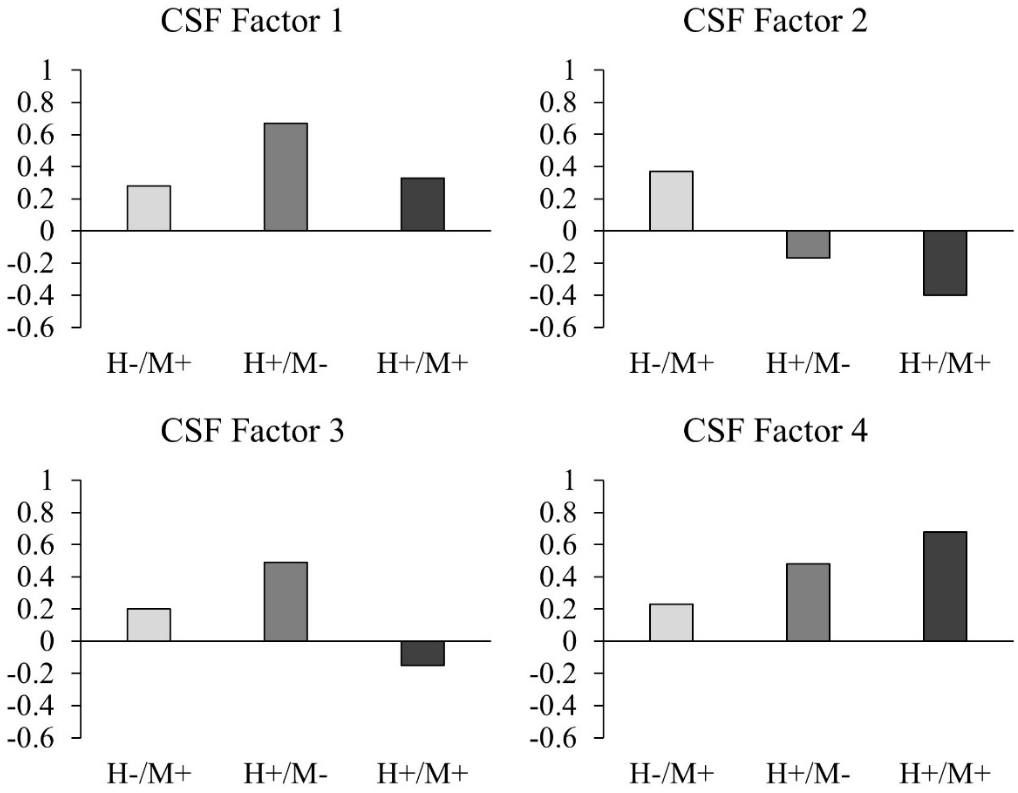

Figure S1. Effect Sizes of Each Group Compared to the HIV-/METH- Group for Each Plasma Factor. Effect sizes (using Cohen's d) were calculated for the HIV-/METH+, HIV+/METH-, and HIV+/METH+ group compared to the control (HIV-/METH-) group for each factor.
